# Joint Effects of Habitat Heterogeneity and Species’ Life-History Traits on Population Dynamics in Spatially Structured Landscapes

**DOI:** 10.1371/journal.pone.0107742

**Published:** 2014-09-18

**Authors:** Xinping Ye, Andrew K. Skidmore, Tiejun Wang

**Affiliations:** 1 Department of Natural Resources, Faculty of Geo-Information Science and Earth Observation (ITC), University of Twente, Enschede, The Netherlands; 2 Foping National Nature Reserve, Shaanxi, China; University of New England, Australia

## Abstract

Both habitat heterogeneity and species’ life-history traits play important roles in driving population dynamics, yet there is little scientific consensus around the combined effect of these two factors on populations in complex landscapes. Using a spatially explicit agent-based model, we explored how interactions between habitat spatial structure (defined here as the scale of spatial autocorrelation in habitat quality) and species life-history strategies (defined here by species environmental tolerance and movement capacity) affect population dynamics in spatially heterogeneous landscapes. We compared the responses of four hypothetical species with different life-history traits to four landscape scenarios differing in the scale of spatial autocorrelation in habitat quality. The results showed that the population size of all hypothetical species exhibited a substantial increase as the scale of spatial autocorrelation in habitat quality increased, yet the pattern of population increase was shaped by species’ movement capacity. The increasing scale of spatial autocorrelation in habitat quality promoted the resource share of individuals, but had little effect on the mean mortality rate of individuals. Species’ movement capacity also determined the proportion of individuals in high-quality cells as well as the proportion of individuals experiencing competition in response to increased spatial autocorrelation in habitat quality. Positive correlations between the resource share of individuals and the proportion of individuals experiencing competition indicate that large-scale spatial autocorrelation in habitat quality may mask the density-dependent effect on populations through increasing the resource share of individuals, especially for species with low mobility. These findings suggest that low-mobility species may be more sensitive to habitat spatial heterogeneity in spatially structured landscapes. In addition, localized movement in combination with spatial autocorrelation may increase the population size, despite increased density effects.

## Introduction

Habitat heterogeneity is increasingly recognized as a significant factor affecting the dynamics and persistence of populations [1]–[5]. For mobile species inhabiting complex landscapes, spatial heterogeneity can influence their behaviour, thereby affecting survival and reproductive performance [6]–[8]. Most frequently, the effect of spatial heterogeneity has been studied either under a so-called binary ‘habitat-matrix’ framework [9]–[11] or under the assumption of stochastic variability wherein local variation is random in space [5], [6]. However, defining a landscape as discrete habitat/matrix patches overlooks the spatial pattern of continuously varying habitat quality that might facilitate or constrain the performance of individuals and therefore population dynamics [12], [13]. In addition, predictions of population dynamics are also influenced by how the spatial variation in habitat quality within a landscape is calculated [14]. In this sense, a continuous representation of habitat quality is expected to more accurately reflect how a species experiences a heterogeneous landscape [15].

One general property of landscape heterogeneity of particular importance for ecological dynamics is its spatial autocorrelation structure, which can be defined as the property of random variables (e.g., habitat quality) taking values over distance that are more (or less) similar than expected for randomly associated pairs of observations due to geographic proximity [16]–[18]. The spatial autocorrelation in habitat quality is known to affect species persistence, for instance, increased spatial autocorrelation could reduce the persistence of small populations [19]–[21]. Yet the effect of habitat spatial structure on population dynamics has rarely been tested for species in spatially structured landscapes. Bolker [4] incorporated spatially correlated heterogeneity into simulation models of sessile organisms and found that spatial autocorrelation generally improves population viability. For mobile species, however, the performance of a population may become different if spatial autocorrelation occurs within the range of dispersal [22], [23]. From an ecological standpoint, the effects of habitat spatial structure on mobile species depend not only on the scale of spatial autocorrelation, but also on how this scale relates to the species movement capacity and habitat requirements [1], [23]. If the scale of spatial autocorrelation is low, the likelihood of an individual encountering a different environment will increase quickly as it moves away from its present location [22]. If the movement range is large, the population becomes well-mixed and hence spatially unstructured [24], [25]. Moreover, species differ in their environmental tolerance, thereby exhibiting differential responses to habitat conditions [26]–[28]. Therefore, we anticipate that habitat spatial structure will interact with species’ environmental tolerance and movement capacity in determining species’ demographic performance as well as population dynamics [29], [30]. For instance, spatial autocorrelation in habitat quality may lead to spatial aggregation of individuals, thereby affecting local population density or competitive pressure on individuals [31]–[33]. Under such circumstances, individual-level variation can also provide underlying mechanisms of population regulation [34]. However, analytical models do not take into account the complexity of the multiple concurrent interactions (e.g., competition between individuals) which may influence population dynamics [35], [36]. The key to improve understanding of the role of habitat spatial structure in driving the population dynamics of mobile species is therefore to link individual behaviours to population phenomena in a spatially explicit modelling framework [12], [37], [38].

In this paper, we explored how habitat spatial structure interact with species’ environmental tolerance and movement capacity to affect the population dynamics of mobile species using a spatially explicit agent-based modelling approach. We compared the responses of four virtual species to four landscape scenarios differing in the scale of spatial autocorrelation in habitat quality. The ecological motivation for this simulation was to understand the combined impacts of these factors on mobile species that are dispersal limited and experience habitat heterogeneity (e.g., animals in mountainous forests). We hypothesized that for species in landscapes with small-scale spatial autocorrelation, the movement-distance does not have a strong effect on population dynamics, whereas for species in landscapes with large-scale spatial autocorrelation, short distance movement would increase the viability of the population.

## Methods

### The Agent-Based Model

The present spatially explicit, agent-based model simulates the population dynamics of a single virtual species in a spatially structured landscape. The description of the model follows the ODD protocol (overview, design concepts, and details) for describing individual- and agent-based models proposed by Grimm *et al.* [39], [40]. The model was implemented in the R environment [41] with the package ‘gstat’ [42].

#### Purpose

The purpose of this model is (i) to understand how habitat spatial structure (depicted by the scale of spatial autocorrelation in habitat quality) influences the population dynamics of mobile species, and (ii) to explore how species’ life-history traits shape the population responses in relation to habitat spatial structure. Since this is a purely theoretical study, the model simulates population performance of four hypothetical species that differ in aspects of life-history traits (low-tolerance vs. high-tolerance; low-mobility vs. high-mobility), rather than predict the population dynamics of any specific species. In other words, the model’s formulation tends to increase generality in a trade-off for realism and precision.

#### Entities, state variables, and scales

The model contains two types of entities: individuals and landscape. The landscape is a grid of 30×30 habitat cells, in which each cell represents a discrete space of the environment, and is characterised by spatial coordinates and habitat quality (*Q*; range 0–1), where habitat quality simply reflects resource conditions essential for an individual’s performance. Cell size is not spatially specified, but is assumed to fulfil the space requirement of an individual. The simulated landscape is, therefore, a continuous representation of habitat quality rather than a binary mosaic of habitat and matrix. The individuals are characterized by the following state variables: identity number, age, spatial location (cell coordinates), resource share, reproduction rate, mortality rate, and moving distance per time step. Only females are simulated in the model, a strategy common to many population models [11], [12]. No age-structure is included in the model. Values of the demographic parameters (see Table 1) are selected in order to meet reasonable assumptions about the hypothetical species (i.e. long-lived animals which generally have low mortality rate and high probability of reproductive success), thereby ensuring this generic model to generate plausible projections. Time runs in discrete steps and the simulation lasts 100 time-steps. The length of a time step is not explicitly specified, but is assumed to be long enough for each individual to accomplish all demographic processes (i.e., movement, reproduction, and mortality).

**Table 1:** Variables, parameters, and initial conditions used in the model.

Parameter	Symbol	Values
Landscape size	-	30×30 cells
Habitat quality of cells	*Q*	0–1
Mean habitat quality of landscape	-	0.5
Scale of spatial autocorrelation in habitat quality	*S*	0.01, 2, 4, 8
Individual’s resource share	*F*	0–1
Birth probability	*B*	0.7
Basic mortality rate	*M*_0_	0.15
Level of environmental tolerance	*C*_envir_	2, 3
Mean moving distance	*D*_mean_	1, 4 cells
Litter size	-	1
Max. lifespan	-	10 time steps
Initial population size	-	100
Length of simulation period	-	100 time steps

#### Process overview and scheduling

**Figure 1:**
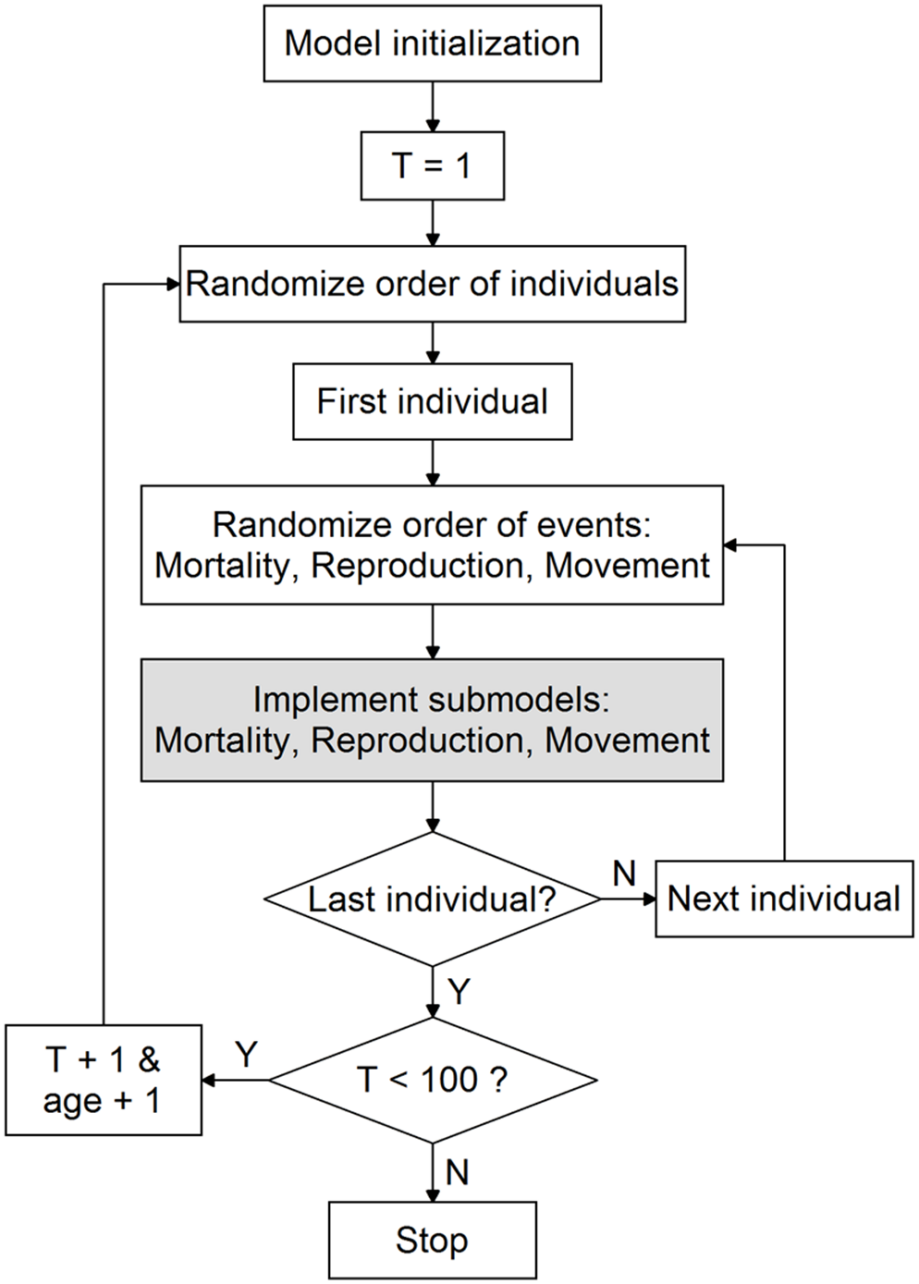
Flow diagram of the main routine used in the simulation model.

**Figure 2:**
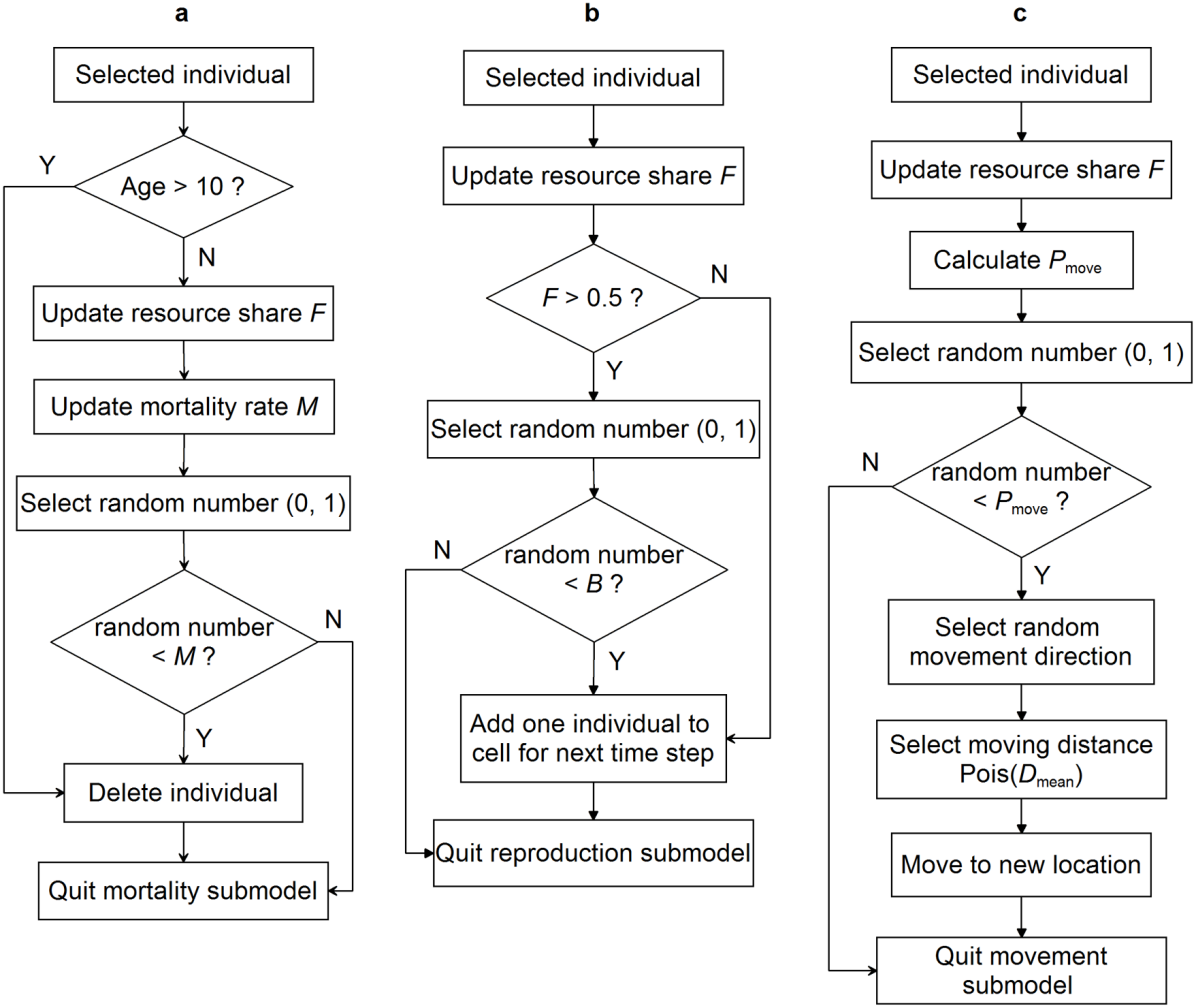
Flow diagrams of the submodels in the simulation model that determine mortality (a), reproduction (b), and (c) movement for a single individual within a time step.

Individuals may move, reproduce, and/or die at each time step. The order of individuals is randomized per time step, and the order of the three events (i.e. move, reproduce, and/or die) is randomized for each individual per time step. Probabilities of mortality, reproduction, and movement of an individual per time step depend on its resource share, which is updated before each event to take into account changes in the cell’s density due to mortality and movement of other individuals within the time step. Only individuals with a resource share beyond 0.5 are capable of reproducing. The newly produced offspring join the population only at the end of the time step, and become adults upon commencement of the following time-step. The model is illustrated in Figures 1 and 2.

#### Design concepts

Population dynamics emerge from individual and species traits. Individuals’ life cycles and movements are imposed by stochastic rules.

Individuals within the same cell affect each other through the “scramble competition” [43] that the resource share (*F*) per individual declines with an increasing number of individuals within a cell. Therefore, an individual’s resource share is determined by habitat quality of the inhabited cell and crowding in that cell, i.e., 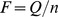, where *Q* is the habitat quality of the cell, and *n* is the number of individuals in the cell. Direct interaction between individuals of different cells is not considered in the model.

All modelled processes are implemented stochastically via a random order of individuals in the main routine, a random order of events, and moving in a random direction. Movement, reproduction, and mortality events are implemented based on probabilities.

Population dynamics are quantified by recording the following variables throughout the last 30 time steps of each run: 1) population size; 2) mean resource share of individuals; 3) mean mortality rate of individuals; 4) proportion of individuals in high-quality cells (*Q*≥0.5); and 5) proportion of individuals experiencing competition. By using the proportion of individuals under different conditions (e.g., residing in high-quality cells and/or experiencing competition), the absolute number of individuals are transformed to a range 0–1, allowing direct comparison between population dynamics of different species, and thereby correcting for differences in their population sizes. For model analysis, each response variable is averaged over the last 30 time steps of each run.

There are no external environmental variables that drive the internal dynamics of the model.

#### Initialisation

At the beginning of each run, a two-dimensional landscape with 30×30 grid cells is generated using an unconditional Gaussian variogram model (*sill* = 0.025, *nugget* = 0, and *range* = *S*), where the *range* parameter defines the scale of spatial autocorrelation in habitat quality. The mean habitat quality of the landscape is fixed at 0.5. After generating a landscape, one hundred individuals are distributed at random through the landscape. The resource share of each individual is calculated based on the habitat quality and crowdedness of the occupied cell. Individuals’ ages are independently drawn from the uniform distribution in the interval (0, lifespan), because preliminary tests based on different distributions (e.g., Poisson distribution) revealed that different initial age structure of individuals had no effect on population dynamics after the first 30 time-steps (see Text S1).

#### Mortality submodel

**Figure 3:**
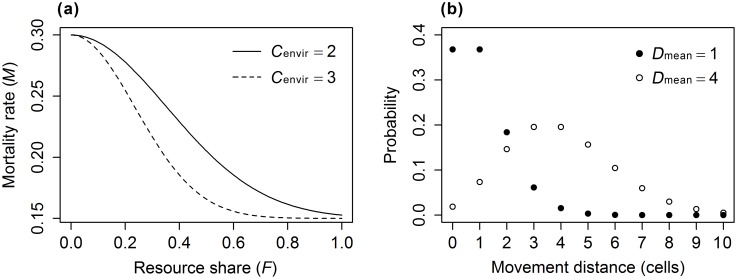
Movement distance distributions (a) and mortality rate functions (b) used to define hypothetical species in the model. *C*_envir_ = 2 and *C*_envir_ = 3 represent low-tolerant and high-tolerant species, respectively. *D*_mean_ = 1 and *D*_mean_ = 4 represent low-mobility and high-mobility species, respectively.

Probabilities of mortality are individual-specific and depend on their resource share per time step. An individual’s mortality rate is calculated as: 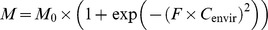, where 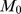 is the basic mortality rate, *F* is the resource share of the individual, and 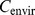 is the level of environmental tolerance. In biological terms, 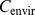 regulates the sensitivity of individual mortality rate to environmental variability (Figure 3a). Individuals that reach the maximal lifespan are “killed” from the population.

#### Reproduction submodel

Only adult individuals with a high resource share (i.e., *F*≥0.5) can reproduce, giving birth to one offspring with a constant probability of *B*. More complex relations, e.g., making reproduction probability and number of offspring function of habitat quality, would by virtue of Jensen’s inequality prohibit comparison between landscapes [15], [44]. The newly produced offspring are attached to their mothers (i.e., in the same cells), and join the population at the end of the current time step. They become adults able to move and reproduce at the following time step.

#### Movement submodel

Individual movement is a conditional response to local habitat quality and crowdedness, i.e., the condition-dependent dispersal [25], [45], and the probability of movement per time step is calculated as: 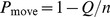, where *Q* is the habitat quality of the cell, and *n* is the number of individuals in the cell. Ecologically, it means that individuals residing in cells of low habitat quality and/or high density tend to depart the cell, while those residing in cells of high quality and/or low density are more likely to stay in the current cell. The movement direction per time step is randomly determined, and the movement distance per time step is drawn from a Poisson distribution with mean 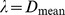 (Figure 3b). The assumption of random walk seems to be unrealistic for species that may actively search the landscape, but is conservative in the sense that effects of spatial heterogeneity are easy to detect [46]. From the moving individuals’ perspective, the landscape is “wrapped”, meaning that an individual that crosses the edge of the landscape continues in the same direction on the opposite edge [47].

### Simulation Experiments

**Figure 4:**
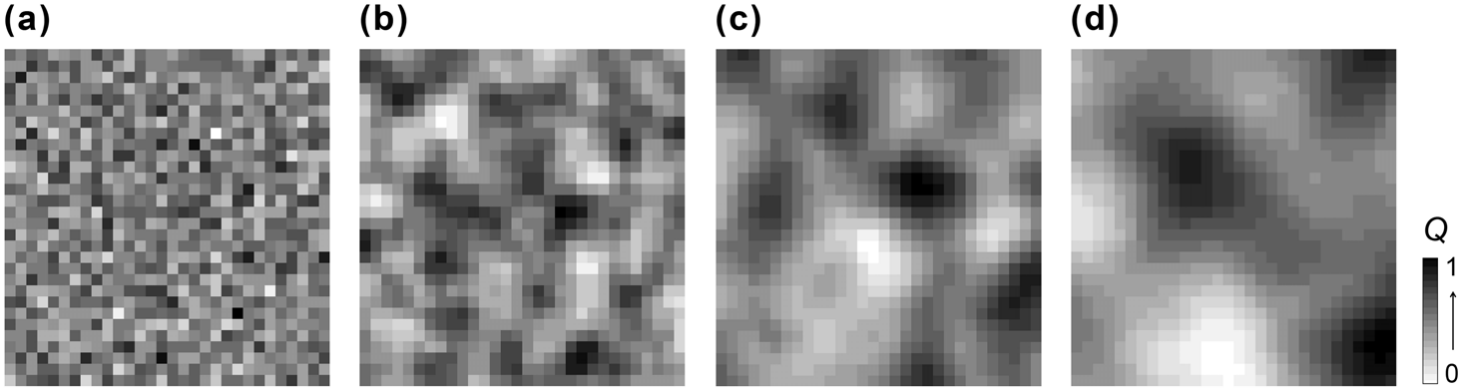
Examples of artificial landscapes differing in the scale of spatial autocorrelation in habitat quality *S*. (a) nearly random, *S* = 0.01; (b) small-scale, *S* = 2; (c) intermediate-scale, *S* = 4; and (d) large-scale, *S* = 8. The mean habitat quality of the landscapes is fixed at 0.5.

To investigate how spatial autocorrelation of habitat quality interacts with species traits to affect population dynamics, we defined four landscape scenarios that reflect different levels of spatial autocorrelation in habitat quality: apparently uncorrelated (*S* = 0.01), small-scale (*S* = 2), intermediate-scale (*S* = 4), and large-scale (*S* = 8) spatial autocorrelation in habitat quality (Figure 4). For each landscape scenario, we compared the population dynamics of four virtual species differing in the level of environmental tolerance (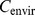 = 2 or 3; Figure 3a) and mean movement distance (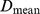 = 1 or 4 cells; Figure 3b). The combination of landscape scenarios and species classes resulted in a total of 16 treatments (4×4 = 16) in the experiments. For each treatment, we conducted 50 replicates, each lasting for 100 time steps. This is based on preliminary runs using 200 time-steps, in which we found that any population reached a state of dynamic equilibrium within the first 50 time-steps.

The flexibility of agent-based models means that they typically have a large number of parameters. Ideally, parameter values would be estimated from empirical data. Unfortunately, such data are scarce, and the number of parameters is so large that it is not possible to investigate every combination of parameter values. Therefore, following a previous sensitivity analysis (data not shown), parameter values were chosen arbitrarily to yield an overall rate of population increase of ∼1 over the first 50 time steps for the species with 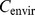 = 2 and 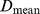 = 1 in landscapes with apparently uncorrelated habitat quality (i.e., landscape scenario *S* = 0.01). Real-world species would experience life-history trade-offs in demographic and movement rates which influence population growth [48]. To permit a more direct comparison of the combined impacts of changing life-history traits and habitat spatial structure, we assumed that all virtual species had the same basic mortality rate (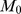) and birth probability (*B*). Since we were interested in combining landscape spatial autocorrelation with species traits, these assumptions were considered acceptable. The detailed parameter values are given in Table 1.

### Statistical Analysis

We first performed a Shapiro-Wilks normality test to examine data normality and found that data were normally distributed. To assess the main effects and interactions among the three experimental factors (i.e., scale of spatial autocorrelation in habitat quality *S*, species’ environmental tolerance 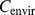, and mean moving distance 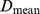), we estimated the magnitude of effects of the variance sources using a three-way analysis of variance (ANOVA), as suggested by White et al. [49]. We performed three-way ANOVA for each of the five response variables (cf. *Observation* section). The three experimental factors were treated as categorical variables in accordance with the experimental design. The effect sizes of the factors and interactions were measured by percentage of variance explained [50] and generalized eta-squared [51], [52] (see Text S2 for the calculation). For each treatment, we also investigated Spearman correlations of the mean resource share of individuals with the proportion of individuals in high-quality cells and with the proportion of individuals experiencing competition. By doing so, we aimed at increasing our understanding of the mechanisms behind observed population dynamics in different landscape scenarios. All data analyses were conducted in the R environment with the ‘ez’ package [53].

## Results

**Figure 5:**
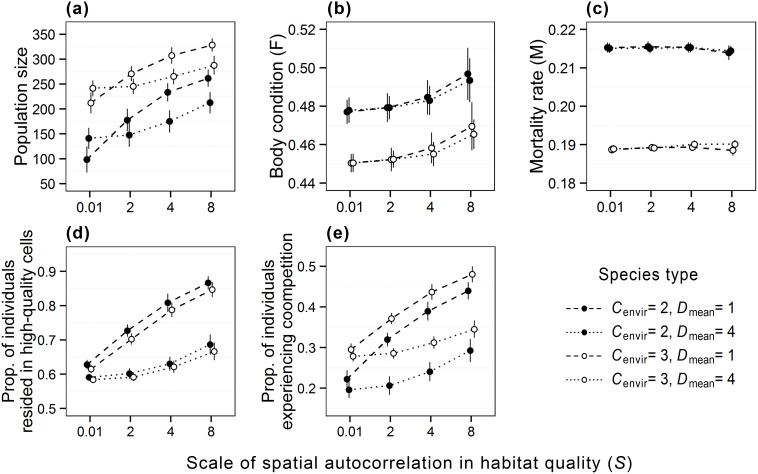
Population responses of four hypothetical species to the scale of spatial autocorrelation in habitat quality *S*. (a) population size; (b) mean resource share of individuals; (c) mean mortality rate of individuals; (d) proportion of individuals in high-quality cells (*Q*≥0.5); (e) proportion of individuals experiencing competition. The figure shows mean±1 SD for 50 replicates for each variable.

All hypothetical species exhibited a constant increase in population size as the scale of spatial autocorrelation in habitat quality increased (Figure 5a). However, the pattern of the increase in population size varied greatly between the species with different movement capacities. Low-mobility species (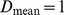) displayed a logarithmic-like increase in response to increased spatial autocorrelation, contrasting with the exponential-like increase of high-mobility species (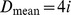). Under the same level of species’ environmental tolerance, low-mobility species showed greater population size than high-mobility species in all landscape scenarios except for the scenario of *S* = 0.01 (Figure 5a).

The analysis of variance revealed that both species’ environmental tolerance and the scale of spatial autocorrelation in habitat quality had strong effects on population size, accounting for ∼47% and ∼33%, respectively, of the variation in population size (Table 2, also see Table S1). Their interaction also had a significant effect (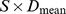 *F*_7,792_ = 487.96, *p*<0.001), but accounted only for ∼1% of the variation in population size (Table 2). Species’ movement capacity and its interaction with scale of spatial autocorrelation in habitat quality also had appreciable effects and explained ∼10% of the variation in population size (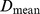 *F*_1,798_ = 23.04, *p*<0.001; 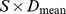 *F*_7,792_ = 85.29, *p*<0.001; Table 2).

**Table 2:** Summary of the percentages of the variation in response variables explained by factors scale of spatial autocorrelation in habitat quality (*S*), species environmental tolerance (*C*_envir_), and mean moving distance (*D*_mean_). Detailed ANOVAs for each response variable are presented in Table S1.

Source	Explained variation (%) in response variable				
	Populationsize	Mean resourceshare of individuals	Mean mortalityrate of individuals	Prop. of individualsin high-quality cells	Prop. of individualsexperiencing competition
*S*	33.16	15.35	0.05	41.18	38.57
*C*_envir_	46.92	62.41	98.96	0.67	13.37
*D*_mean_	2.81	0.16	0.01	44.60	34.13
*S*×*C*_envir_	1.09	0.01	0.05	0.04	0.44
*S*×*D*_mean_	7.01	0.26	0.04	9.48	7.88
*C*_envir_×*D*_mean_	0.03	0.01	0.01	0.04	0.29
*S*×*C*_envir_×*D*_mean_	0.02	0.01	0.00	0.02	0.05
Total variation explainedby the full model (*R* ^2^)	0.91	0.78	0.99	0.96	0.95

All virtual species also showed an exponential increase in mean resource share as the scale of spatial autocorrelation in habitat quality increased (Figure 5b), and low-tolerant species had appreciably higher mean resource shares than high-tolerant populations (Mann–Whitney test, *p*<0.01). Species’ environmental tolerance had the greatest effect on individual resource share, explaining ∼62% of the variation in mean resource share (Table 2, also see Table S1). Scale of spatial autocorrelation in habitat quality accounted for ∼15% of the variation in mean resource share of individuals (Table 2). In addition, the effect of movement capacity on mean resource share was appreciably greater when habitat quality was spatially autocorrelated at a broad scale (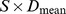 *F*_7,792_ = 21.18, *p*<0.001).

For the mean mortality rate of individuals, species’ environmental tolerance had a dominant effect and accounted for ∼99% of the variation (Table 2, also see Table S1). In contrast, the scale of spatial autocorrelation in habitat quality and species’ movement capacity had little effect on the mean mortality rate of individuals, due to the dominant effect of species’ environmental tolerance (Figure5c and Table 2).

**Figure 6:**
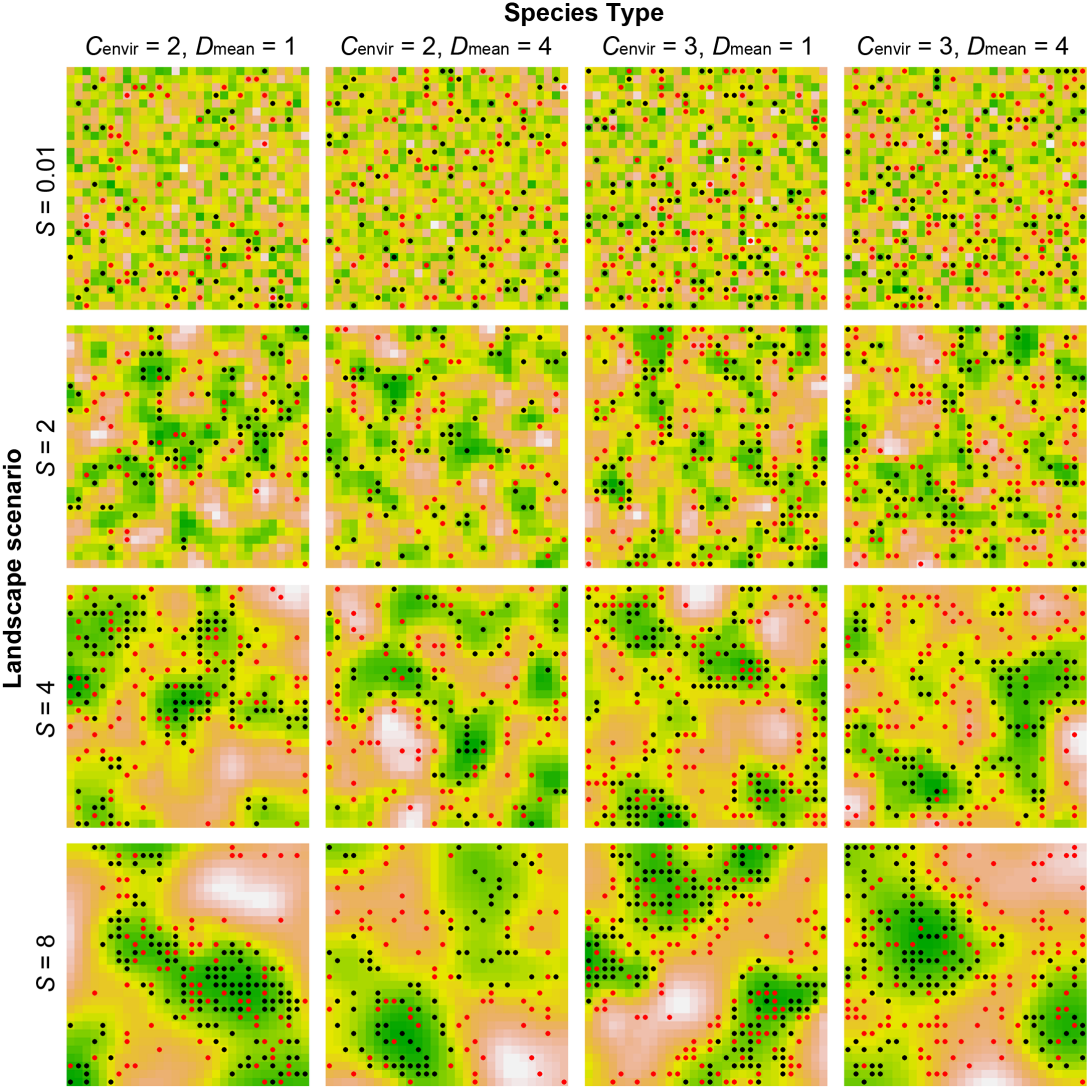
Sample patterns of spatial distribution of individuals under different landscape scenarios. Hypothetical species are parameterized by environmental tolerance *C*_envir_ and mean moving distance *D*_mean_. Black dots represent individuals residing in cells of *Q*≥0.5 and free of competition, while red dots are individuals residing in cells of *Q*<0.5 or experiencing competition. Greener colour indicates higher habitat quality.

The proportion of individuals residing in high-quality cells and the proportion of individuals experiencing competition reflect the spatial distribution of individuals in response to the spatial structure of habitat quality (Figure 6, also see Figures S1 and S2). Increasing the scale of spatial autocorrelation in habitat quality increased the proportion of individuals residing in high-quality cells, especially for low-mobility species (Figure 5d, also see Figure S1). Species’ movement capacity and the scale of spatial autocorrelation in habitat quality both had strong effects, accounting for ∼45 and 41%, respectively, of the variation in the proportion of individuals in high-quality cells (Table 2, also see Table S1). Their interaction also had a significant effect and explained ∼9% of the variation (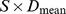 *F*_7,792_ = 2273.5, *p*<0.001).

Likewise, the proportion of individuals experiencing competition increased as the scale of spatial autocorrelation in habitat quality increased, and low-mobility species had higher proportions when habitat quality was spatially autocorrelated (Figure5e, also see Figure S2). The scale of spatial autocorrelation in habitat quality had the greatest effect on the proportion of individuals experiencing competition, followed by species’ movement capacity and environmental tolerance (Table 2, also see Table S1). The interaction between the scale of spatial autocorrelation in habitat quality and species’ movement capacity also had a significant effect, accounting for ∼8% of the variation in the proportion of individuals experiencing competition (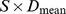 *F*_7,792_ = 469.54, *p*<0.001).

**Figure 7:**
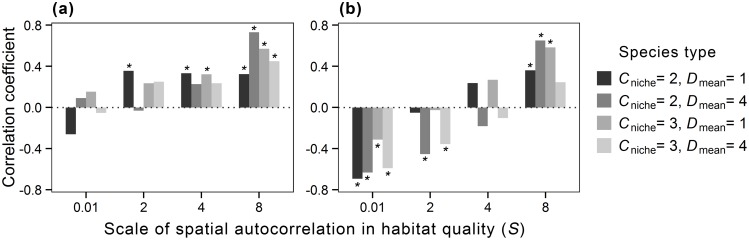
Coefficients of Spearman correlations of the mean resource share of individuals with (a) the proportion of individuals in high-quality cells and (b) the proportion of individuals experiencing competition for four hypothetical species under different landscape scenarios. Bars marked with an asterisk (*) indicate the coefficients are statistically significant at *P*<0.05.

In general, the mean resource share of individuals was positively associated with the proportion of individuals in high-quality cells when habitat quality was spatially autocorrelated at a broad scale (Figure 7a). In contrast, the associations between mean resource share of individuals and the proportion of individuals experiencing competition were differential, depending on the spatial structure of habitat quality. When the spatial structure of habitat quality shifted from spatially random to autocorrelated at a broad scale, the correlation changed from negative to positive (Figure 7b), indicating that local movement in combination with spatial autocorrelation can actually increase population size and resource share despite of increased density effects.

## Discussion

In this study, we explored how habitat spatial autocorrelation interacts with species’ life-history attributes to influence population dynamics within spatially structured landscapes. We found that increasing the scale of spatial autocorrelation in habitat quality can greatly increase the population size as well as mean resource share of virtual species and in line with common expectation, high-tolerant species had an appreciably higher population size than low-tolerant species. These results are generally consistent with previous findings that more specialized species are more affected by habitat spatial heterogeneity [4], [8], [14], [54]. These observed patterns may be partly explained by edge effects, which is one of the factors influencing population dynamics [55]. As the scale of spatial autocorrelation increases, habitat quality becomes less rugged across space, forming fewer but larger suitable areas in the landscape, and consequently reduces changes in abundance or fitness of low-tolerant species with respect to low quality of “edge” areas. In contrast, the high-tolerant species, due to higher tolerances to variation in habitat quality, may be insensitive to such edge effects [48]. Moreover, our results are comparable to the population dynamics in the “source-sink” context. Spatial autocorrelation in habitat quality can result in neighbourhoods of high-quality and low-quality areas, thereby creating source-sink dynamics in relation to habitat heterogeneity [31], [32]. If a landscape is spatially autocorrelated (particularly at large extents), individuals dispersing away from “source” habitats are more likely to encounter higher quality habitats rather than disperse into a demographic sink.

Species’ movement capacity can interact with habitat heterogeneity to influence population dynamics and distribution of individuals [56], [57]. Our simulation demonstrated that low-mobility species had greater population sizes than high-mobility species in the presence of spatial autocorrelation in habitat quality. The findings also suggest that species’ movement capacity plays an important role in shaping the increase in population size in response to an increased spatial autocorrelation in habitat quality, where low-mobility species had a logarithmic-like increase in response to increased scale of spatial autocorrelation, contrasting with the exponential-like increase for high-mobility species. This may be because, under the assumption of random walk, individuals with short-distance movement are likely to stay in suitable habitats, while long-distance movement will increase the risk of landing in an unsuitable habitat when habitat quality is autocorrelated at fine scales [4], [58], [59]. Such contrasting patterns of population increase in relation to movement capacity indicate that the effect of distance-based movement capacity may function in highly scale-dependent ways, and its relative strength is determined by interactions among movement distance, the scale of spatial autocorrelation, and the effects of species competition [56], [57], [60]. For instance, the logarithmic-like increase in population size of low-mobility species in response to increased spatial autocorrelation indicates that the benefit from short-distance movement was buffered by increasing effects of population density, whereas the exponential-like increase in population size for high-mobility species may suggest that long-distance movement will reduce competitive interactions between individuals, thereby increasing the net population. Skelsey and Garrett [61] predicted that the magnitude of dispersal (number of individuals) should be maximized at intermediate scales of heterogeneity, i.e., when the scale of spatial heterogeneity is neither too fine nor too coarse relative to the movement capacity of a species. This potentially provides an additional explanation for the observed patterns in population dynamics in response to spatial autocorrelation in habitat quality.

The population dynamics of species inhabiting complex landscapes generally involve two major components: the dispersal of individuals and habitat-specific mortality rates [30]. The interplay between spatial autocorrelation in habitat quality and species life-history traits can lead to spatial aggregation of individuals, creating variation in demographic performance among individuals [31], [32]. This is evident by our model, where the proportions of individuals in high-quality cells and the proportion of individuals experiencing competition increased differentially in response to increased spatial autocorrelation between species with different movement capacity (see Figure S1 and Figure S2). These results indicate that localized movement in combination with habitat spatial structure may increase the population size despite of increased density effects in the presence of spatial autocorrelation in habitat quality. Furthermore, we found that the mean resource share was positively correlated with the proportion of individuals experiencing competition when habitat quality was spatially autocorrelated. This may sound contradictory, because an individual’s resource share would be, by definition, reduced by increased local crowding. However, increasing spatial autocorrelation in habitat quality could also improve the quality of individuals by increasing the probability of finding high-quality habitat within the range of movement, which may buffer the density-dependent effects on the population [21]. Such complex relationships indicate how the interplay of movement capacity and environmental autocorrelation can jointly influence the outcome of competitive interactions [62].

The results of this simulation are contingent upon numerous simplifying model assumptions, and it remains to be seen whether the same or similar effects also occur for species living in real landscapes. For example, the model used a simplified landscape with the edge being “wrapped” so that an individual crossing the edge of the landscape continues in the same direction on the opposite edge [47]. However, moving individuals would experience sharp discontinuities as they cross the edges if the simulated landscape boundaries are not periodic. To avoid this, one can generate landscapes with either periodic boundary conditions or with a larger spatial extent, depending on the study of interest [63]. Furthermore, we assumed individuals move in a random manner. The random movement assumption seems to be unrealistic for species that may actively search the landscape within their home range (e.g. birds and mammals). However, if individuals can perceive and orient new habitat location from some distance, it may result in a reduced effect of habitat spatial structure on population dynamics. Therefore, the random movement assumption is conservative in the sense that effects of landscape heterogeneity are easy-to-detect [46]. Moreover, male individuals were not modelled based on the perspective that females form the reproductive unit and thereby determine the population dynamics [11], [12]. We also excluded life-history trade-offs in demographic and movement rates by assuming that only mortality rate is associated with local habitat quality and density. However, more complex relations, e.g., making both mortality and reproduction functions of habitat quality, would increase the unpredictability in model outputs and prohibit comparison between landscapes due to the Jensen’s inequality [15], [44].

Although the model incorporates numerous simplifying assumptions, the general prediction that low-mobility species will have greater sensitivity to changing scale of spatial autocorrelation in habitat quality strongly suggests that mechanisms shaping population dynamics are complex and depend on both habitat spatial heterogeneity and species’ life history traits considered. We have yet to confirm them in the field, but if the spatial autocorrelation favouring species exists, we may expect a nonlinear relationship between local population dynamics and habitat heterogeneity in the sense that species movement limitation represents a threshold [22]. Nevertheless, understanding which mechanisms in spatially structured landscapes regulate a population is important for explaining species abundances and designing management plans for species conservation.

## Supporting Information

Figure S1 **Sample patterns of spatial distribution of individuals residing in high-quality cells under different landscape scenarios.**
(DOCX)

Figure S2 **Sample patterns of spatial distribution of individuals experiencing competition under different landscape scenarios.**
(DOCX)

Table S1 **Factorial ANOVAs on each of the five response variables under different scale of spatial autocorrelation in habitat quality and species’ environmental tolerance and movement capacity.**
(DOCX)

Text S1 **Testing for the effect of different initial age-structures on model output.**
(DOCX)

Text S2 **Calculation of the generalized eta-squared in factorial ANOVA.**
(DOCX)
