# Supplementary material for: Joint Effects of Habitat Heterogeneity and Species’ Life-History Traits on Population Dynamics in Spatially Structured Landscapes
Source: PLoS One. 2014 Sep 18;9(9):e107742. doi: 10.1371/journal.pone.0107742 (PMC4169469; doi:10.1371/journal.pone.0107742)
Supplement: Figure S2 — Sample patterns of spatial distribution of individuals experiencing competition under different landscape scenarios. (DOCX) [file pone.0107742.s002.docx]

**Figure S2.** Sample patterns of spatial distribution of individuals experiencing competition under the four landscape scenarios. Hypothetical species are parameterized by the level of environmental tolerance *C*_envir_ and mean moving distance *D*_mean_. Black dots represent individuals free of competition, while red dots are individuals experiencing competition. Greener colour indicates higher habitat quality. N is the population size.

|  | ***C*_envir_ = 2, *D*_mean_ = 1** | ***C*_envir_ = 2, *D*_mean_ = 4** | ***C*_envir_ = 3, *D*_mean_ = 1** | ***C*_envir_ = 3, *D*_mean_ = 4** |
| --- | --- | --- | --- | --- |
| **Scenario *S* = 0.01** | 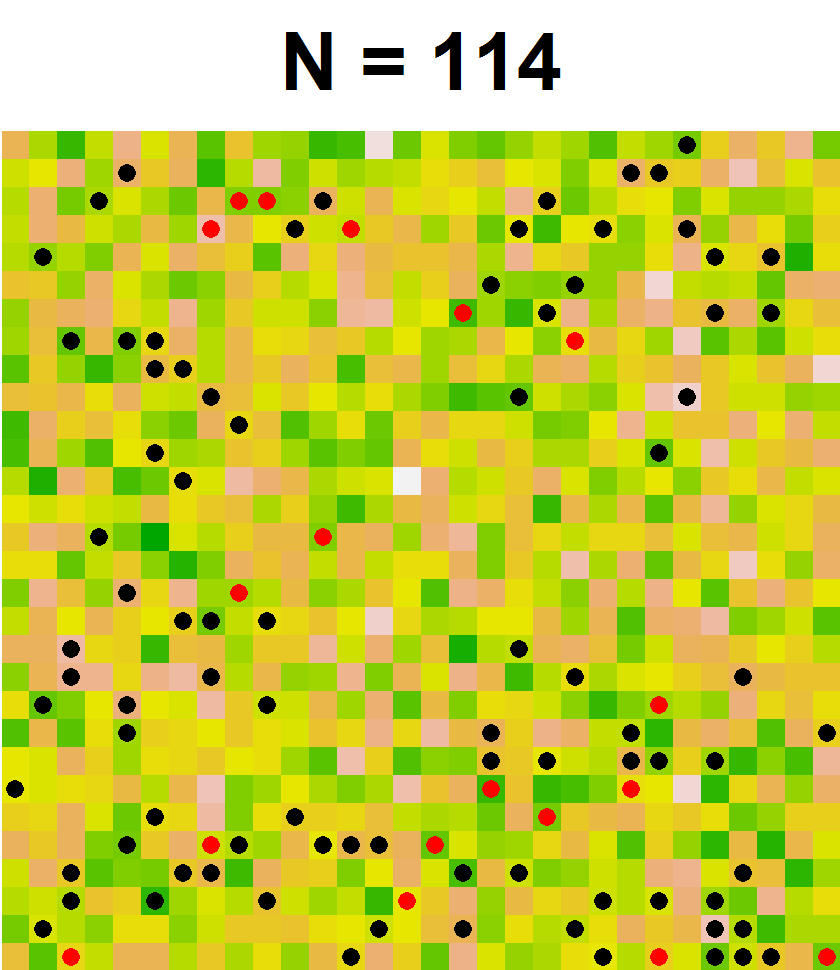 | 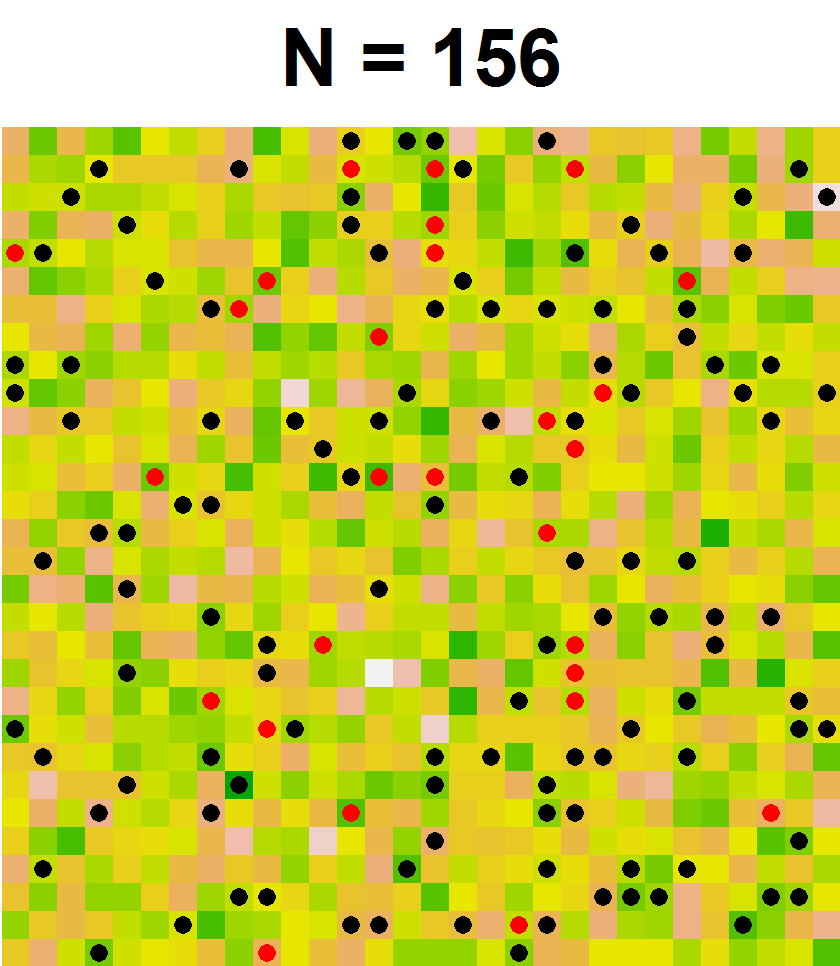 | 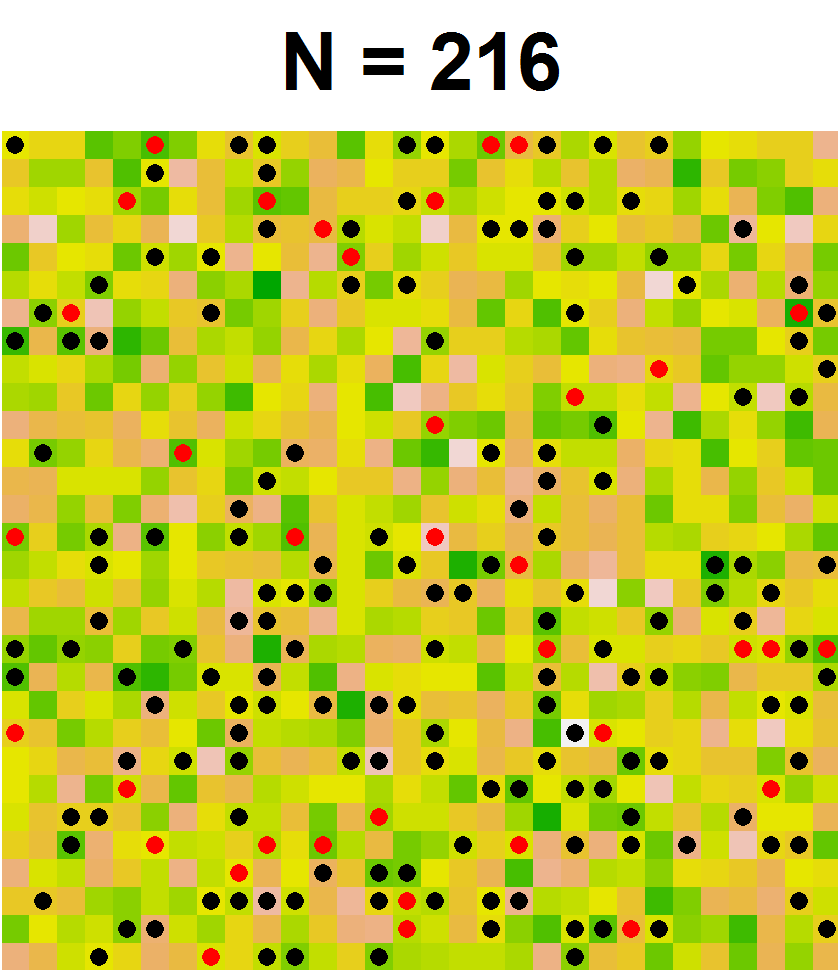 | 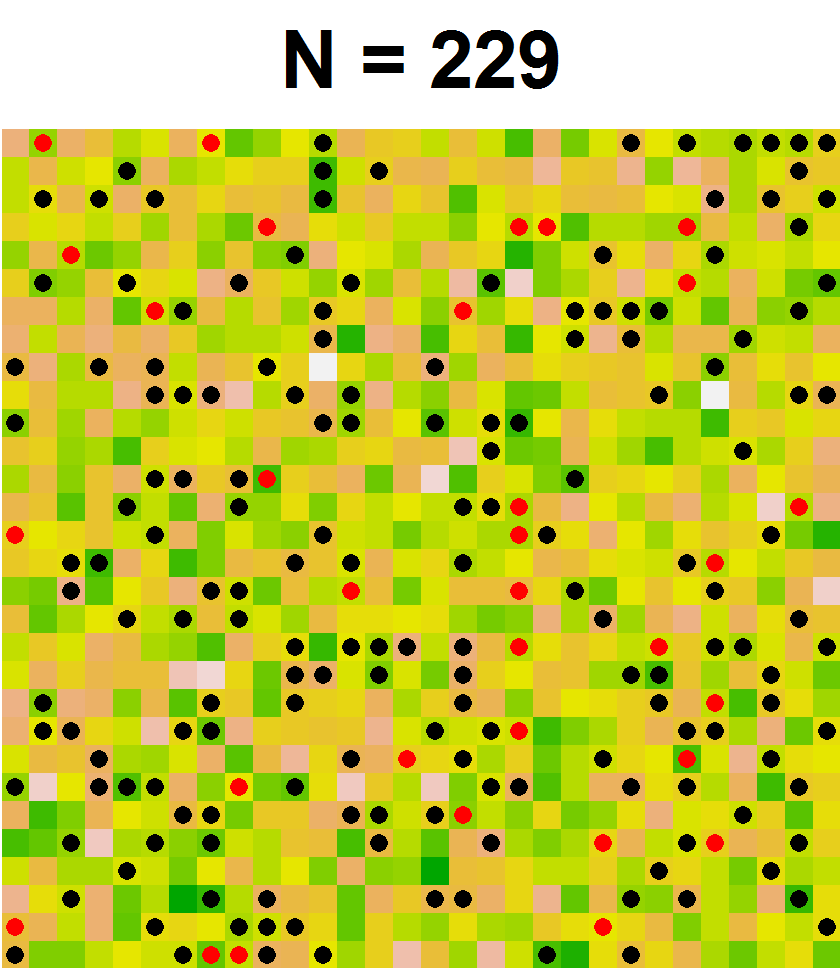 |
| **Scenario *S* = 2** | 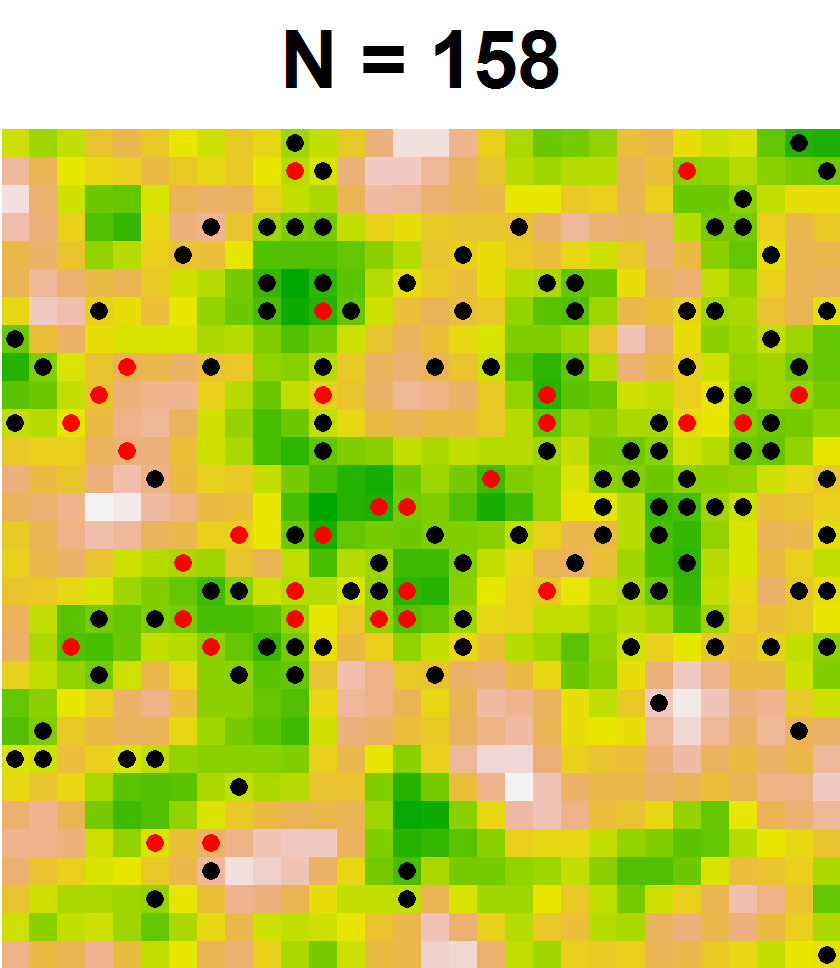 | 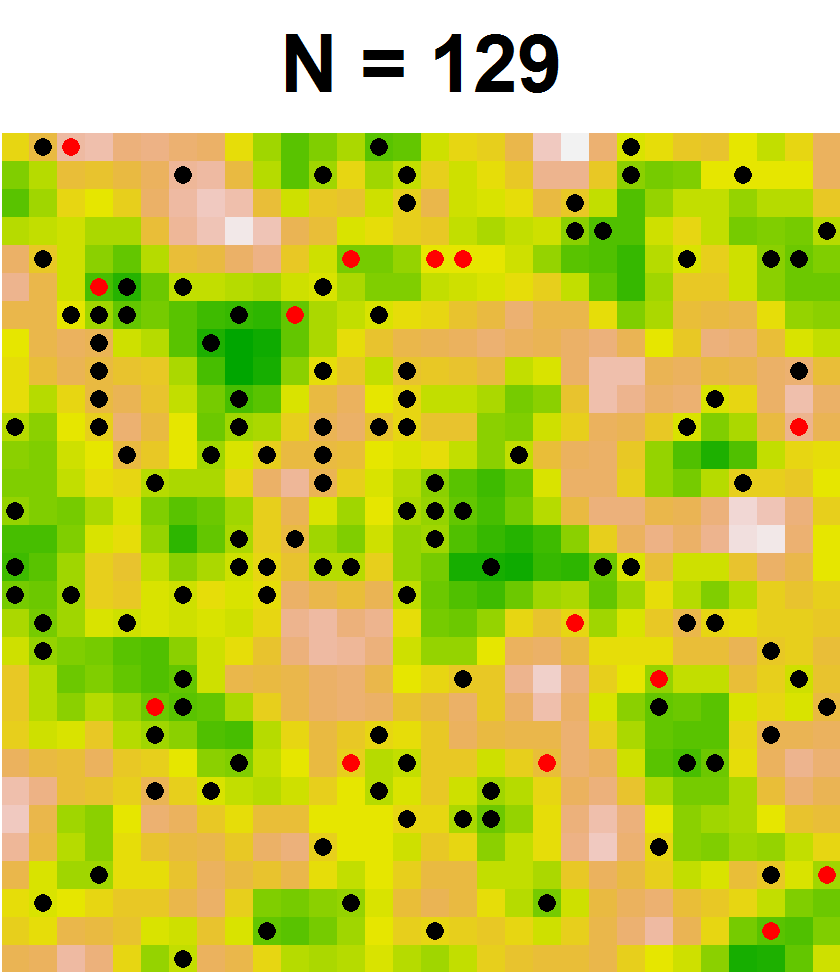 | 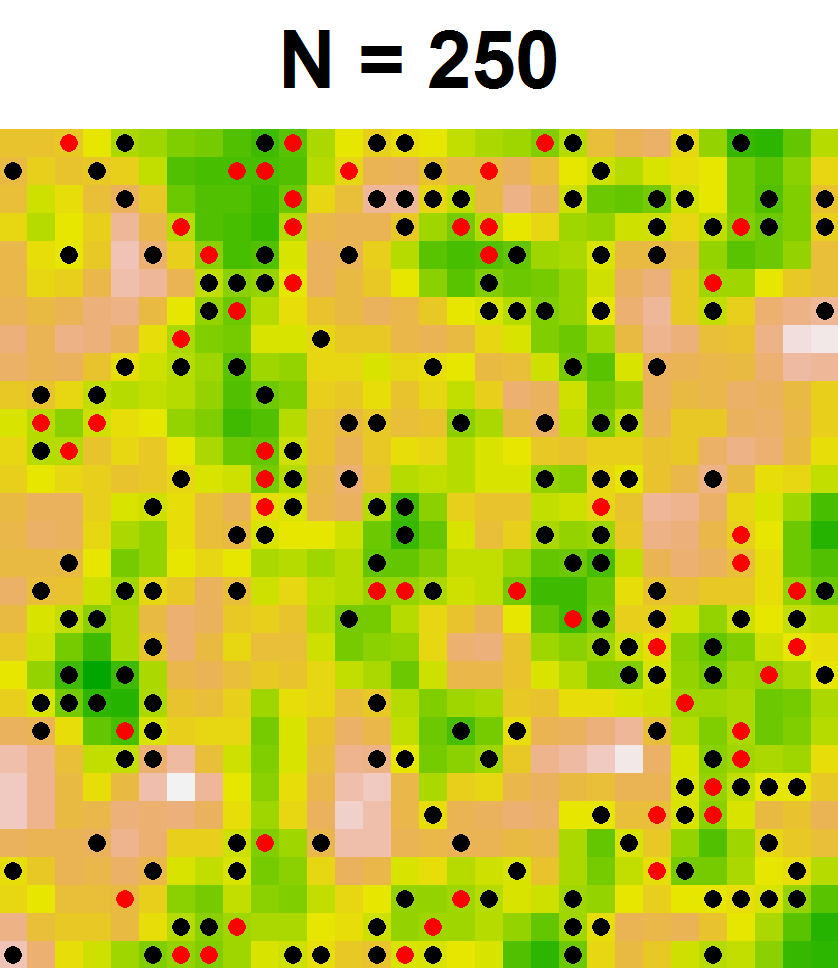 | 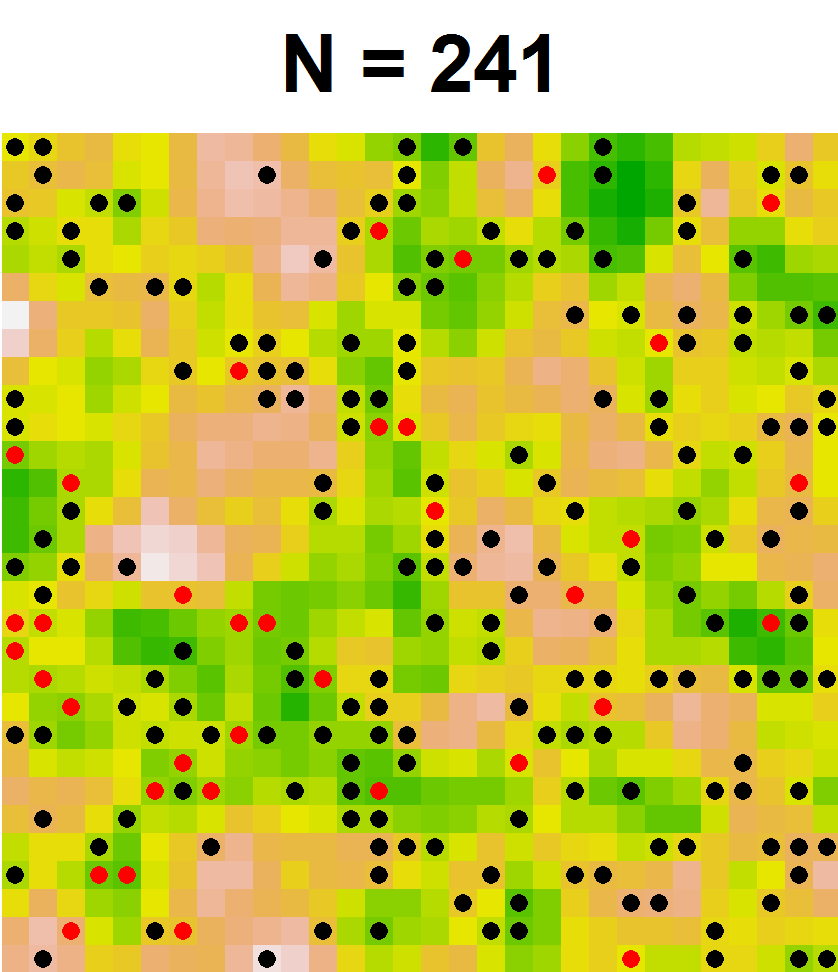 |
| **Scenario *S* = 4** | 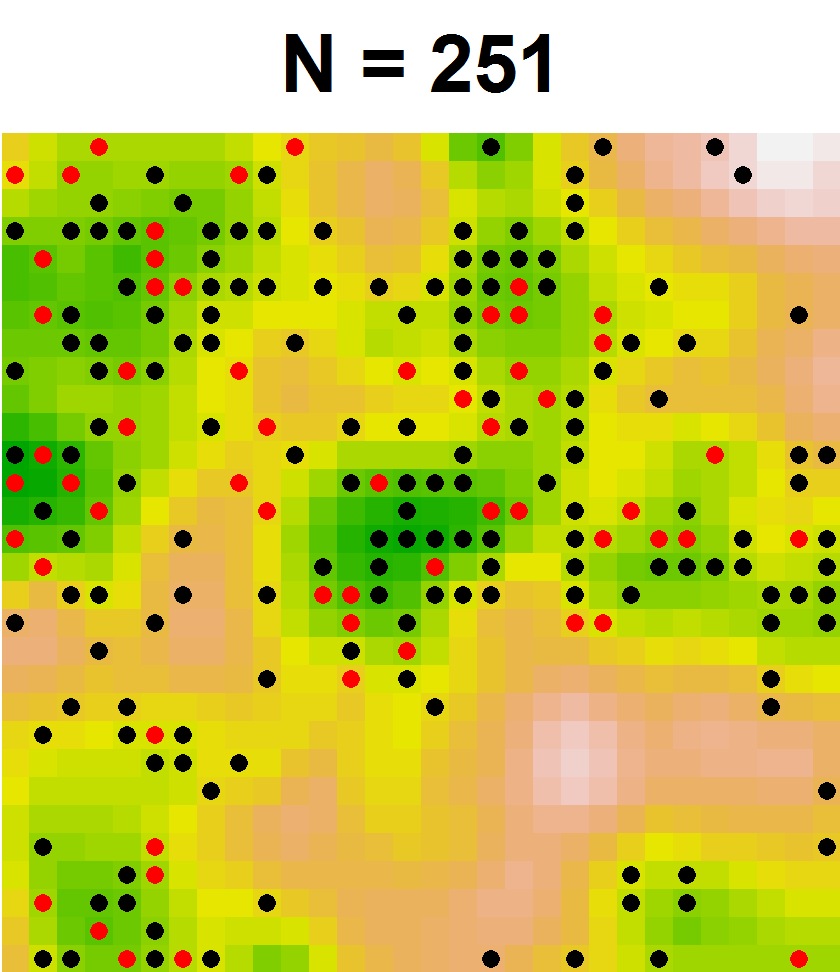 | 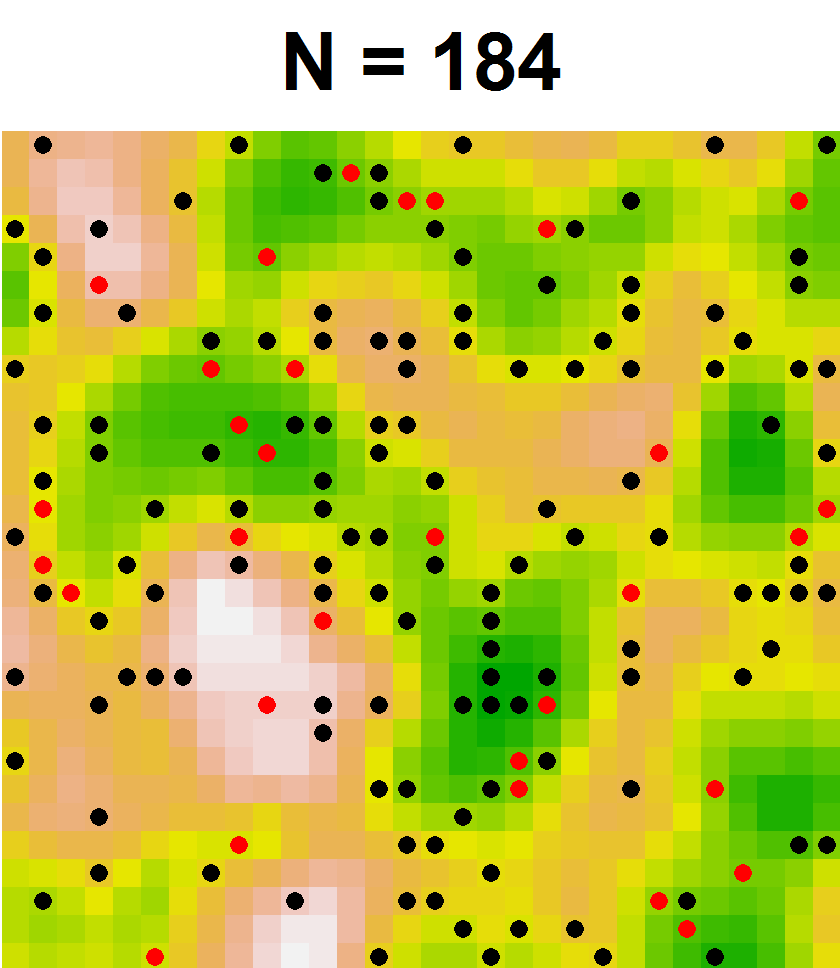 | 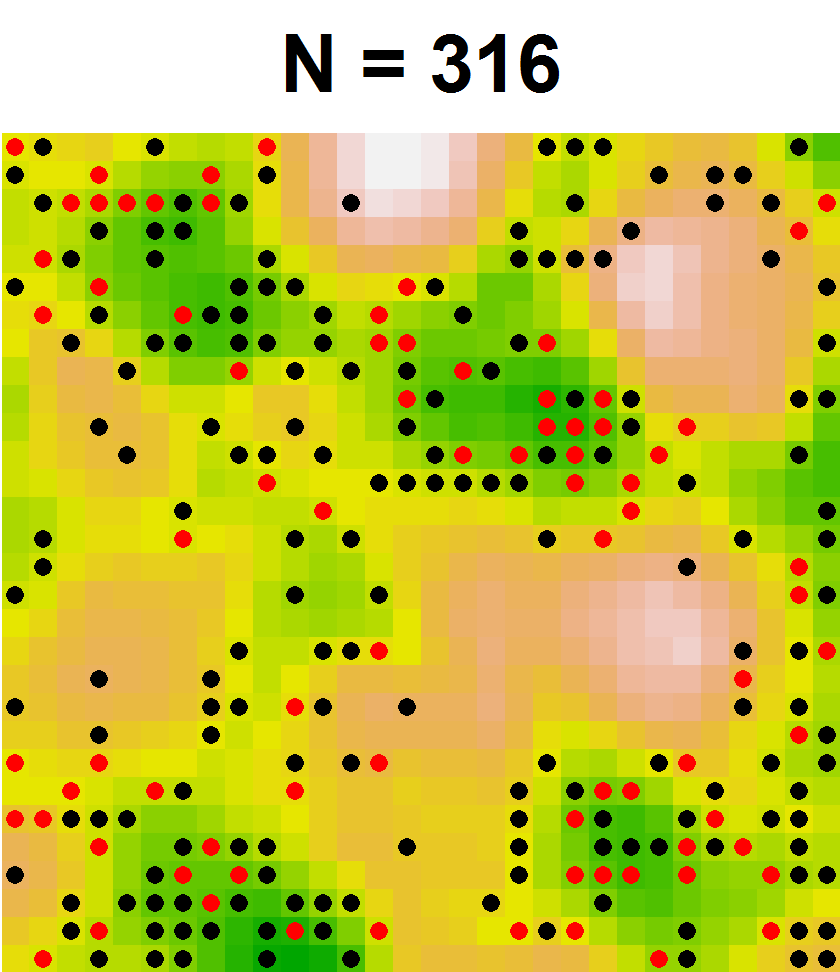 | 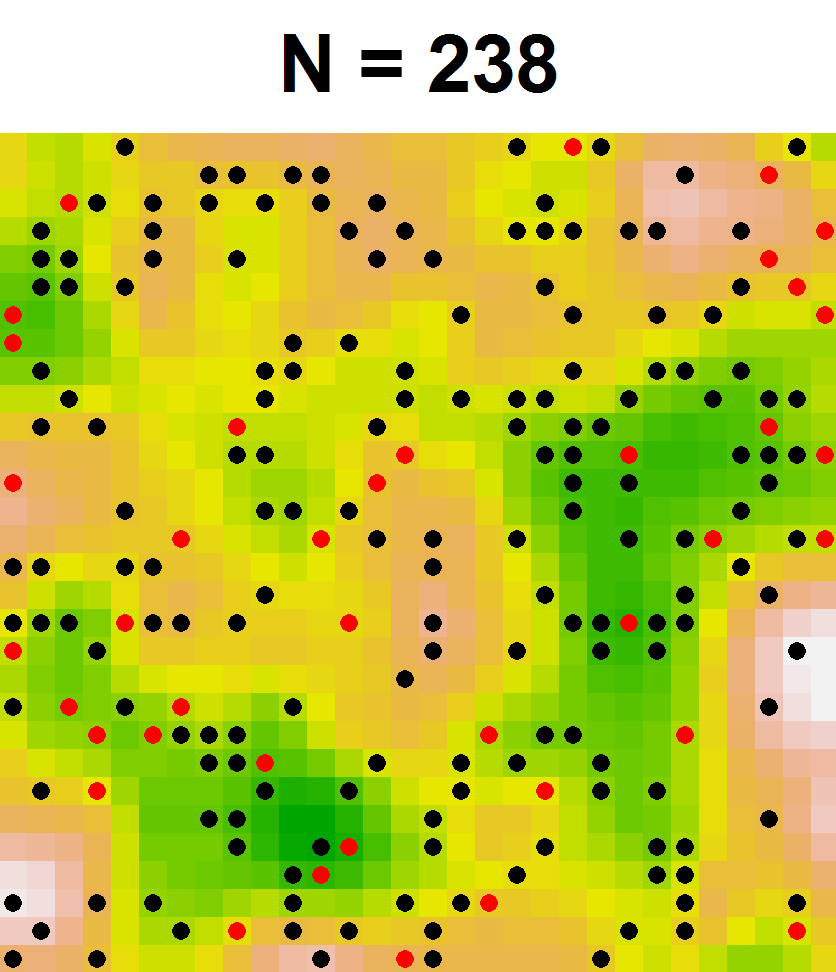 |
| **Scenario *S* = 8** | 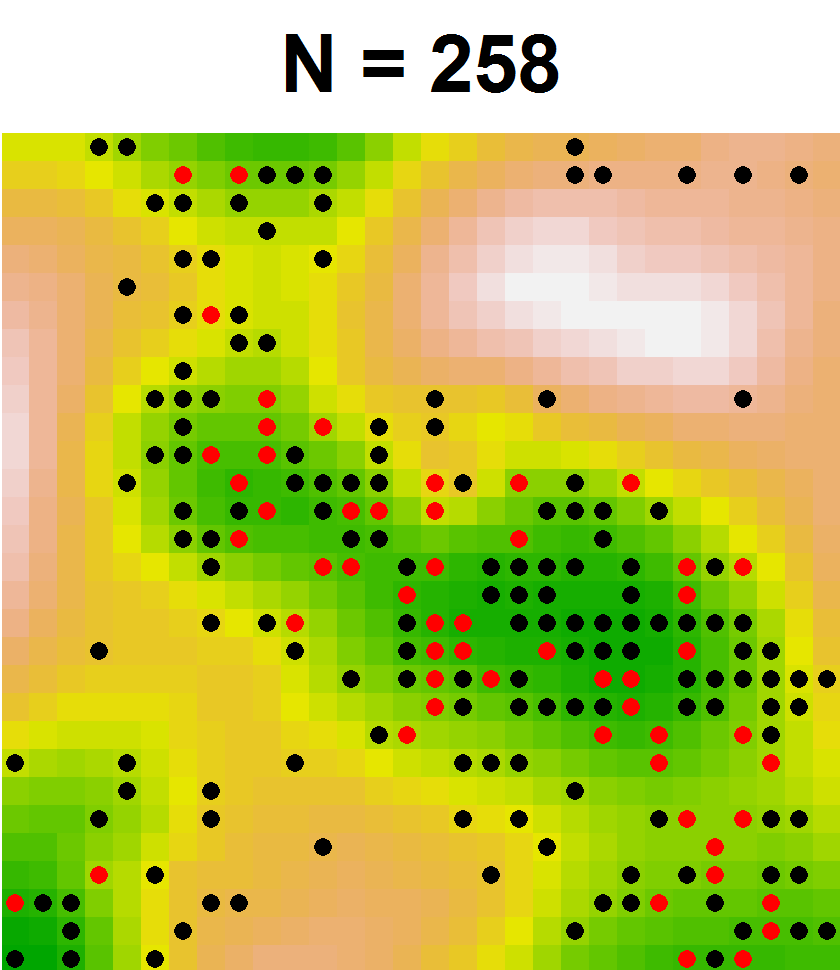 | 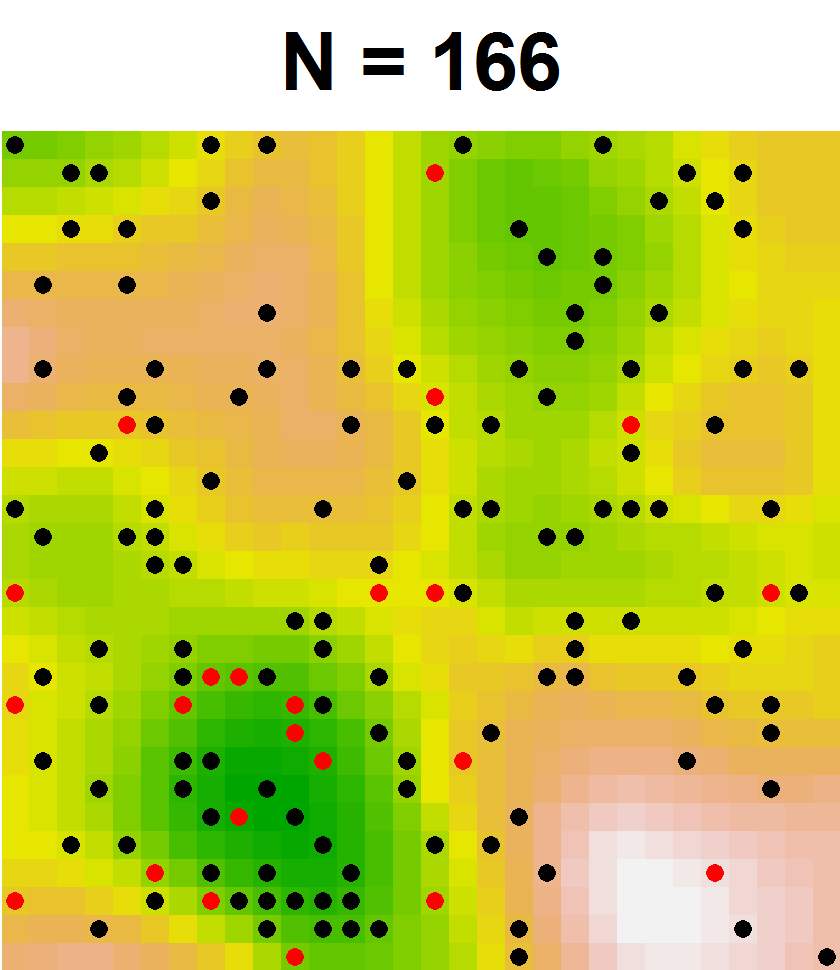 | 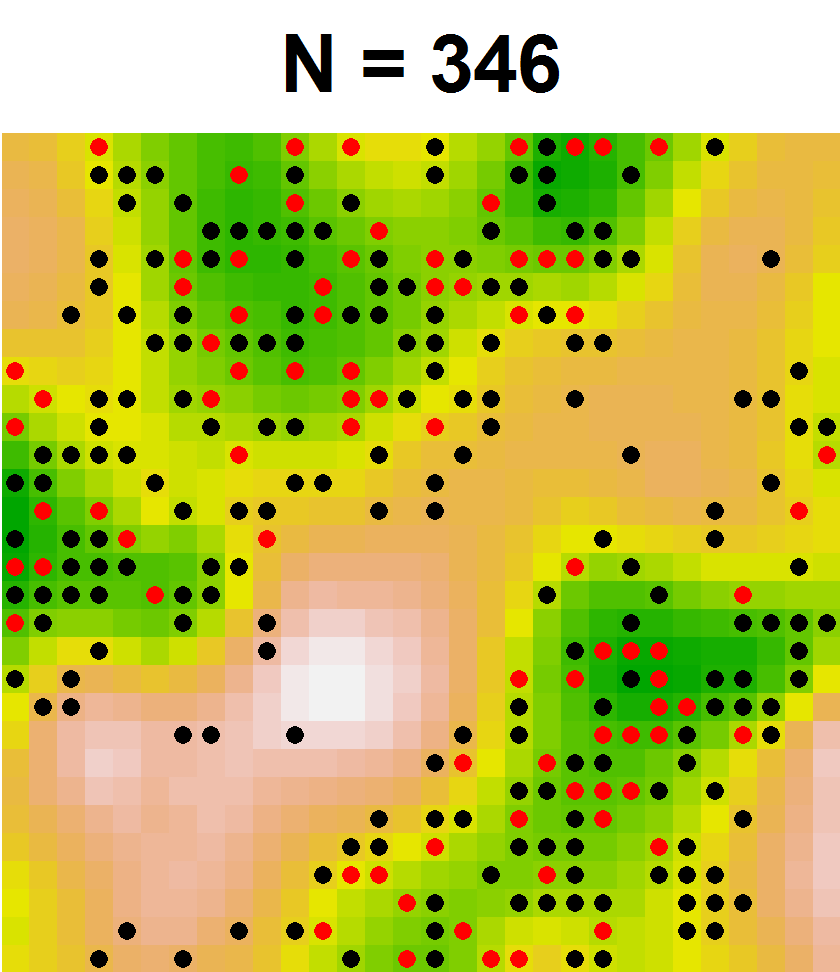 | 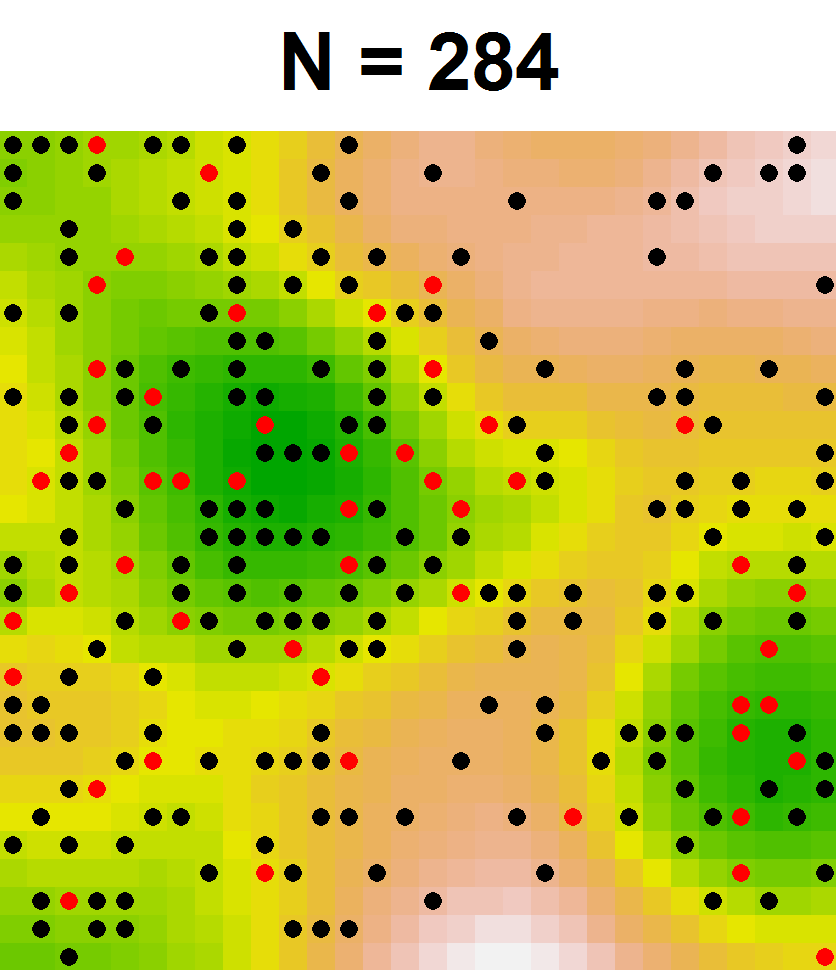 |
