# Supplementary material for: Joint Effects of Habitat Heterogeneity and Species’ Life-History Traits on Population Dynamics in Spatially Structured Landscapes
Source: PLoS One. 2014 Sep 18;9(9):e107742. doi: 10.1371/journal.pone.0107742 (PMC4169469; doi:10.1371/journal.pone.0107742)
Supplement: Table S1 — Factorial ANOVAs on each of the five response variables under different scale of spatial autocorrelation in habitat quality and species’ environmental tolerance and movement capacity. (DOCX) [file pone.0107742.s003.docx]

## Table S1 Factorial ANOVAs on each of the five response variables under different scale of spatial autocorrelation in habitat quality (*S*) and species’ environmental tolerance (*C*_envir_) and movement capacity (*D*_mean_). The *F*-ratio is the variance between groups to the variance within groups. Percentage of *SS*_total_ is defined as the sum of squares of a factor relative to total sum of squares (i.e., *SS*_effect_/*SS*_total_). $\boldsymbol{\eta}_{\mathbf{G}}^{\mathbf{2}}$ is the generalized eta-squared.

| Source of variation | *df* | *F*-ratio | *P*-value | Percentage of *SS*_total_ | $\eta_{G}^{2}$ |
| --- | --- | --- | --- | --- | --- |
| *Population size* | | | | | |
| *S* | 3 | 984.24 | < 0.001 | 33.16 | 0.79 |
| *C*_envir_ | 1 | 4178.39 | < 0.001 | 46.92 | 0.84 |
| *D*_mean_ | 1 | 249.83 | < 0.001 | 2.81 | 0.24 |
| *S* × *C*_envir_ | 3 | 32.42 | < 0.001 | 1.09 | 0.11 |
| *S* × *D*_mean_ | 3 | 208.21 | < 0.001 | 7.01 | 0.44 |
| *C*_envir_ × *D*_mean_ | 1 | 2.32 | 0.128 | 0.03 | 0.00 |
| *S* × *C*_envir_ × *D*_mean_ | 3 | 1.08 | 0.432 | 0.02 | 0.00 |
| Residuals | 784 |  |  | 8.95 |  |
| *Mean resource share of individuals* | | | | | |
| *S* | 3 | 184.02 | < 0.001 | 15.35 | 0.41 |
| *C*_envir_ | 1 | 2244.84 | < 0.001 | 62.41 | 0.74 |
| *D*_mean_ | 1 | 5.88 | 0.016 | 0.16 | 0.01 |
| *S* × *C*_envir_ | 3 | 0.10 | 0.963 | 0.01 | 0.00 |
| *S* × *D*_mean_ | 3 | 3.11 | 0.026 | 0.26 | 0.01 |
| *C*_envir_ × *D*_mean_ | 1 | 0.37 | 0.541 | 0.01 | 0.00 |
| *S* × *C*_envir_ × *D*_mean_ | 3 | 0.10 | 0.961 | 0.01 | 0.00 |
| Residuals | 784 |  |  | 21.80 |  |
| *Mean mortality rate of individuals* | | | | | |
| *S* | 3 | 13.83 | < 0.001 | 0.05 | 0.05 |
| *C*_envir_ | 1 | 88415.67 | < 0.001 | 98.96 | 0.99 |
| *D*_mean_ | 1 | 10.20 | 0.002 | 0.01 | 0.01 |
| *S* × *C*_envir_ | 3 | 14.52 | < 0.001 | 0.05 | 0.05 |
| *S* × *D*_mean_ | 3 | 11.41 | < 0.001 | 0.04 | 0.04 |
| *C*_envir_ × *D*_mean_ | 1 | 13.28 | < 0.001 | 0.01 | 0.02 |
| *S* × *C*_envir_ × *D*_mean_ | 3 | 1.12 | 0.341 | 0.00 | 0.00 |
| Residuals | 784 |  |  | 0.88 |  |
| *Prop. of individuals resided in high-quality cells* | | | | | |
| *S* | 3 | 2715.10 | < 0.001 | 41.18 | 0.91 |
| *C*_envir_ | 1 | 132.65 | < 0.001 | 0.67 | 0.14 |
| *D*_mean_ | 1 | 8820.83 | < 0.001 | 44.60 | 0.92 |
| *S* × *C*_envir_ | 3 | 2.68 | 0.046 | 0.04 | 0.01 |
| *S* × *D*_mean_ | 3 | 625.01 | < 0.001 | 9.48 | 0.71 |
| *C*_envir_ × *D*_mean_ | 1 | 8.65 | 0.003 | 0.04 | 0.01 |
| *S* × *C*_envir_ × *D*_mean_ | 3 | 1.45 | 0.228 | 0.02 | 0.01 |
| Residuals | 784 |  |  | 3.96 |  |
| *Prop. of individuals experiencing competition* | | | | | |
| *S* | 3 | 1914.23 | < 0.001 | 38.57 | 0.88 |
| *C*_envir_ | 1 | 1991.38 | < 0.001 | 13.37 | 0.72 |
| *D*_mean_ | 1 | 5082.07 | < 0.001 | 34.13 | 0.87 |
| *S* × *C*_envir_ | 3 | 21.74 | < 0.001 | 0.44 | 0.08 |
| *S* × *D*_mean_ | 3 | 391.29 | < 0.001 | 7.88 | 0.60 |
| *C*_envir_ × *D*_mean_ | 1 | 42.90 | < 0.001 | 0.29 | 0.05 |
| *S* × *C*_envir_ × *D*_mean_ | 3 | 2.59 | 0.052 | 0.05 | 0.01 |
| Residuals | 784 |  |  | 5.27 |  |
