# Supplementary material for: Joint Effects of Habitat Heterogeneity and Species’ Life-History Traits on Population Dynamics in Spatially Structured Landscapes
Source: PLoS One. 2014 Sep 18;9(9):e107742. doi: 10.1371/journal.pone.0107742 (PMC4169469; doi:10.1371/journal.pone.0107742)
Supplement: Text S1 — Testing for the effect of different initial age-structures on model output. (DOCX) [file pone.0107742.s004.docx]

**Text S1.** Testing for the effects of initial age-structures on model output.

We simulated the dynamics of populations under different initial age-structures: uniform distribution, poisson distribution with λ = 2, and poisson distribution with λ = 5. We found no significant differences among population sizes under different initial age-structures (Kruskal-Wallis test, *p* < 0.05; Figure 1). The age structure of populations at the 50^th^ time step of the simulations under different initial age-structures also showed little difference (Figure 2).


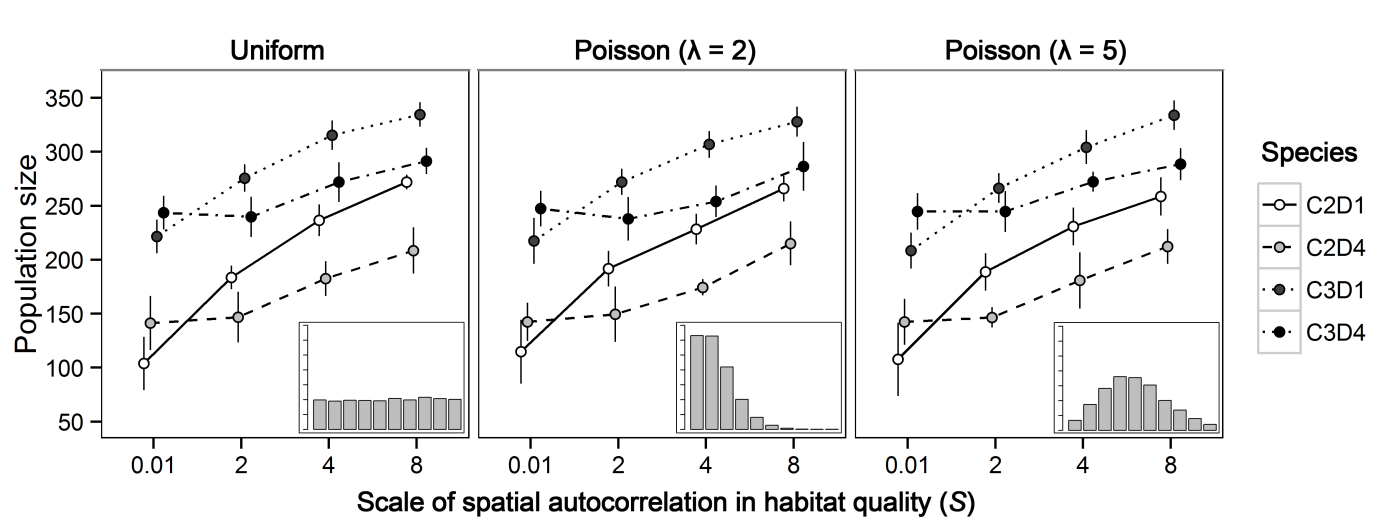


Figure 1. simulated population sizes under different initial age structure distributions: uniform distribution, poisson distribution with λ = 2, and poisson distribution with λ = 5. The inserts are the distributions of initial age distributions of populations.


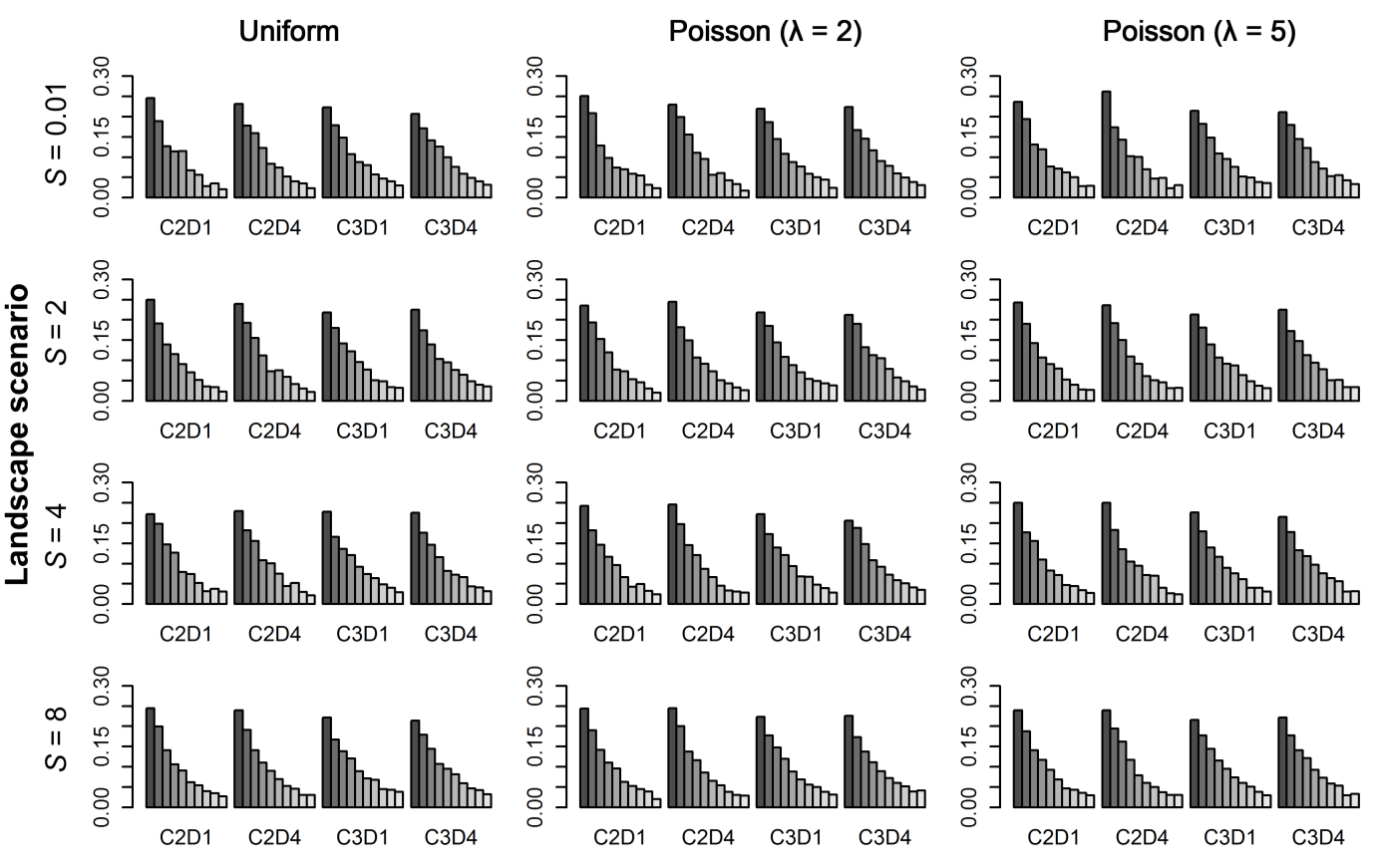


**Figure 2**. Histograms of age of individuals at the 50^th^ time step of the simulation under different initial age-structure distributions: uniform distribution, poisson distribution with λ = 2, and poisson distribution with λ = 5.
