# Supplementary material for: Joint Effects of Habitat Heterogeneity and Species’ Life-History Traits on Population Dynamics in Spatially Structured Landscapes
Source: PLoS One. 2014 Sep 18;9(9):e107742. doi: 10.1371/journal.pone.0107742 (PMC4169469; doi:10.1371/journal.pone.0107742)
Supplement: Text S2 — Calculation of the generalized eta-squared in factorial ANOVA. (DOCX) [file pone.0107742.s005.docx]

## Text S1 Calculation of generalized eta-squared

The generalized eta-squared [1, 2], $\eta_{G}^{2}$, is a measure of effect size that permits comparisons of results across both between-subjects and within-subjects designs. The generalized eta-squared is calculated as:

$$\eta_{G}^{2}=\frac{{SS}_{\mathrm{Effect}}}{\delta\times{SS}_{\mathrm{Effect}}+\sum_{\mathrm{Measured}} {SS}_{\mathrm{Measured}}+\sum_{K} {SS}_{K}}$$

where $\delta$ = 1 if the effect of interest is a manipulated factor and zero otherwise. The index Measured runs over all sources of variance that do not include subjects but do involve a measured factors, (e.g., a blocking factor or a Block × Manipulated factor interaction), and ${SS}_{\mathrm{Measured}}$ is the sum of squares for such an effect. The index K runs over all sources of variance that involve subjects or covariates, and ${SS}_{K}$ is the sum of squares for such a source of variation. ${SS}_{\mathrm{Effect}}$ is the sum of squares for the factor for which the effect size is being estimated.

**Reference**

1. Olejnik S. and Algina J. (2003). Generalized eta and omega squared statistics: measures of effect size for some common research designs. Psychological Methods 8: 434-447.
2. Bakeman, R. (2005). Recommended effect size statistics for repeated measures designs. Behavior Research Methods, 37: 379-384.
